# [Corrigendum] Network pharmacology combined with experimental validation to investigate the effect of Rongjin Niantong Fang on chondrocyte apoptosis in knee osteoarthritis

**DOI:** 10.3892/mmr.2025.13581

**Published:** 2025-05-28

**Authors:** Jun Chen, Ting Zhang, Qingqing Luo, Ruyi Wang, Yuting Dai, Zhenyuan Chen, Chutian Zhang, Xuzheng Chen, Guangwen Wu

Mol Med Rep 29: 102, 2024; DOI: 10.3892/mmr.2024.13226

Following the publication of the above article, the authors have drawn to the Editor's attention that a number of corrections are required to four figures featured in the above article, and a textual change is also required in the Results section. Concerning the textual change, in the last sentence of the first paragraph of the Results on p. 4, “…Schischk and 11 for *Angelica sinensis* (Oliv) Diels”, should have read as: “…Schischk and **10** for *Angelica sinensis* (Oliv) Diels”, as shown correctly in Fig. 1C on p. 5.

Secondly, concerning the flow cytometric (FCM) data shown in the right-hand flow chart in the second row of Fig. 5E, the authors have realized that these data were inadvertently copied across from the third flow chart in the first row of Fig. 4C. After examining their original data, the authors have realized that the second flow chart in the second row of Fig. 5E was misplaced (showing the results from the “siPARP1+H_2_O_2_” experiment), and the corrected version of this figure is presented in this corrigendum (the corresponding statistical graph shown in Fig. 5F has been replaced as well).

Thirdly, concerning the western blot data shown in Fig. 8 on p. 11, the β-actin internal reference proteins pertaining to the cleaved-caspase-3, PARP1 and cleaved-PARP1 experiments were selected incorrectly for this figure. These data, and the corresponding statistical graphs shown in Fig. 8A-D, have been corrected in the new version of Fig. 8 provided in this corrigendum.

Fourthly, the FCM flow charts to represent the “3,200 µg/ml RJNTF” and “PJ34 inhibitor group” experiments in Fig. 9C on p. 12 were wrongly placed, and both these data and the corresponding statistical graphs have also been corrected in the revised version of Fig. 9 included in this corrigendum.

Finally, in Fig. 10 on p. 13, “$” in Fig. 10D (relating to the statistical analysis of the data) should have been written as “ns”, and in the corresponding figure legend, “^$^P<0.05 vs. RJNTF group” should have been written as “^ns^P<0.05 vs. 1,600 µg/ml RJNTF group”. These corrections have been attended to in the revised version of Fig. 10 provided in this corrigendum.

All the authors approve of the publication of this corrigendum, and the authors are grateful to the Editor of *Molecular Medicine Reports* for granting them the opportunity to publish this. The authors regret that these errors were included in the paper, and also apologize to the readership for any inconvenience caused.

## Figures and Tables

**Figure 5.**
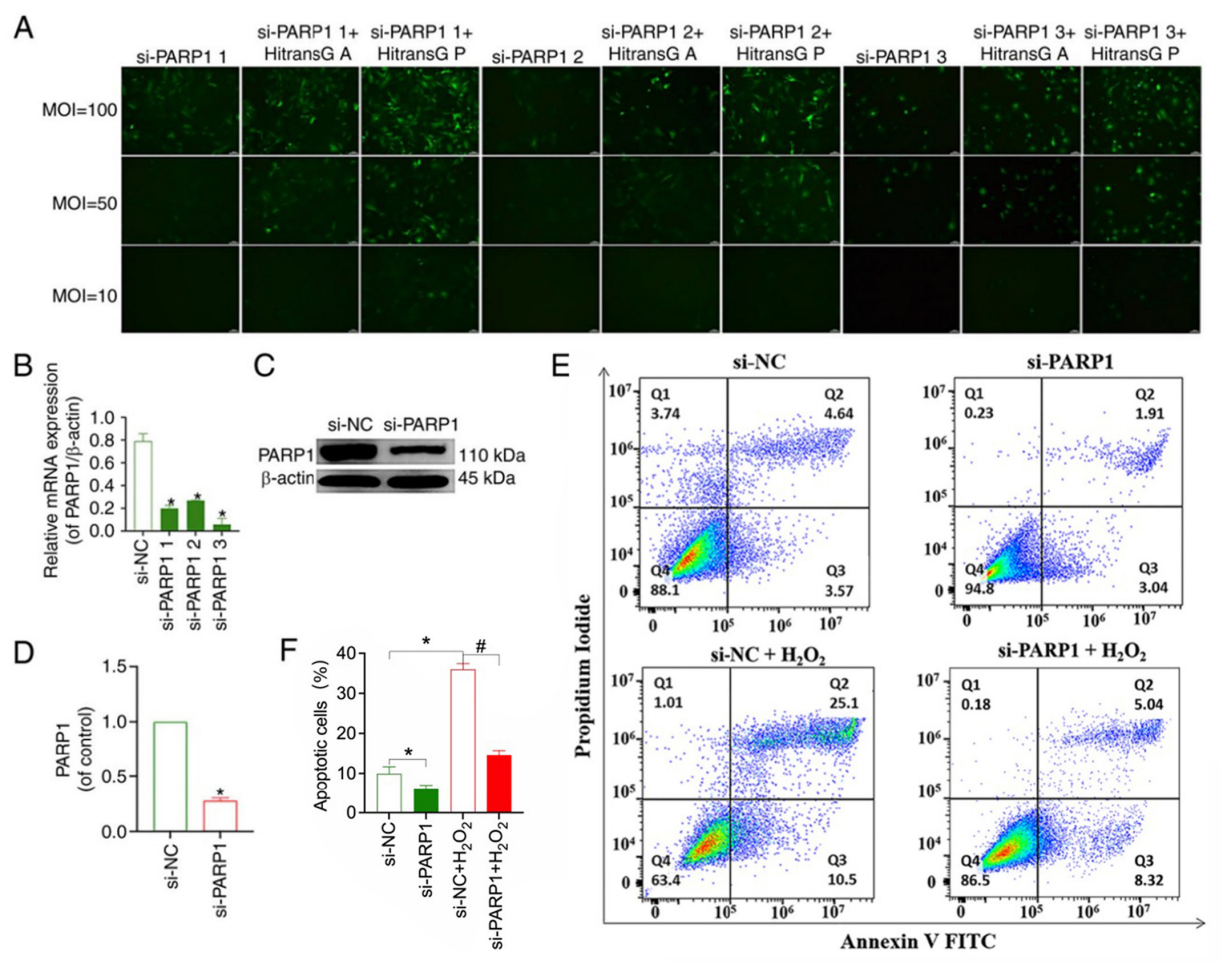
Effect of transfection with a PARP1-knockdown lentivirus on the apoptosis of chondrocytes. (A) eGFP fluorescence intensity under different treatment conditions and transfection with the PARP1-knockdown lentivirus. Magnification, ×100. PARP1 (B) mRNA and (C) protein expression levels after transfection with three different PARP1-knockdown lentiviruses. *P<0.05 vs. si-NC group. (D) Quantitative analysis of the relative protein expression levels of PARP1. *P<0.05 vs. si-NC group. (E) Apoptosis of chondrocytes in groups following PARP1 knockdown. (F) Analysis of chondrocyte apoptosis rate in the different groups. *P<0.05 vs. si-NC group; ^#^P<0.05 vs. si-NC+ H_2_O_2_ group. si, small interfering RNA; NC, negative control; MOI, multiplicity of infection; PARP1, poly [ADP-ribose] polymerase-1; H_2_O_2_, hydrogen peroxide.

**Figure 8.**
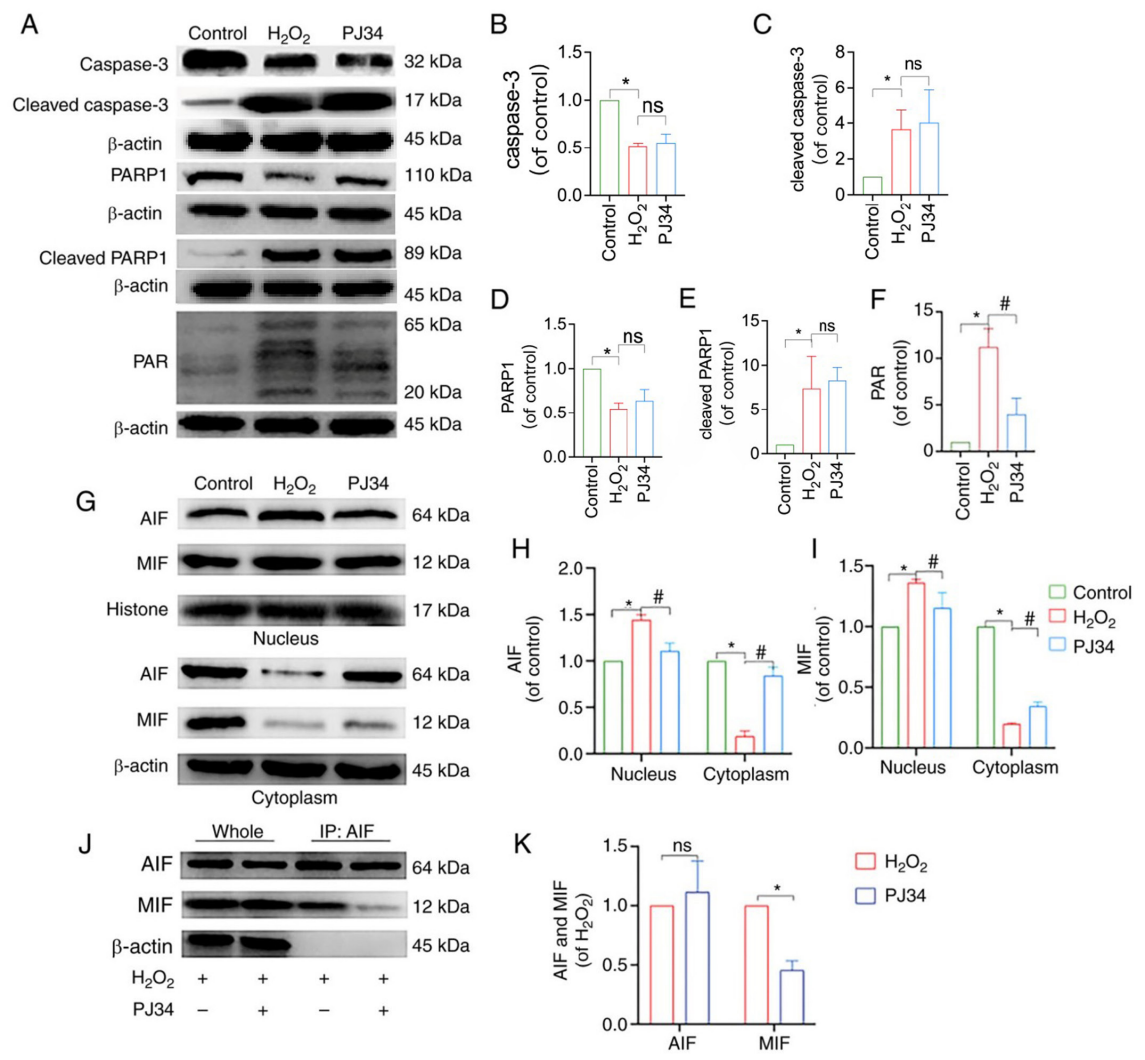
Effect of PJ34-mediated PARP1 inhibition on the expression of PARP1/AIF pathway-related proteins. (A) Protein expression levels of caspase-3, cleaved caspase-3, PARP1, cleaved PARP1 and PAR in chondrocytes in each treatment group. Quantitative analysis of the relative protein expression levels of (B) caspase-3, (C) cleaved caspase-3 (D) PARP1, (E) cleaved PARP1 and (F) PAR. (G) Protein expression levels of AIF and MIF in the nucleus and cytoplasm of the chondrocytes in the different treatment groups. Quantitative analysis of the relative protein expression levels of (H) AIF and (I) MIF. (J) IP analysis of AIF with MIF, where the immunoprecipitate is AIF. (K) Analysis of AIF and MIF protein interactions. *P<0.05 vs. control group; ^#^P<0.05 vs. H_2_O_2_ group. PARP1, poly [ADP-ribose] polymerase-1; PAR, poly ADP-ribose; AIF, apoptosis inducing factor; MIF, migration inhibitory factor; H_2_O_2_, hydrogen peroxide; ns, not significant; IP, immunoprecipitation.

**Figure 9.**
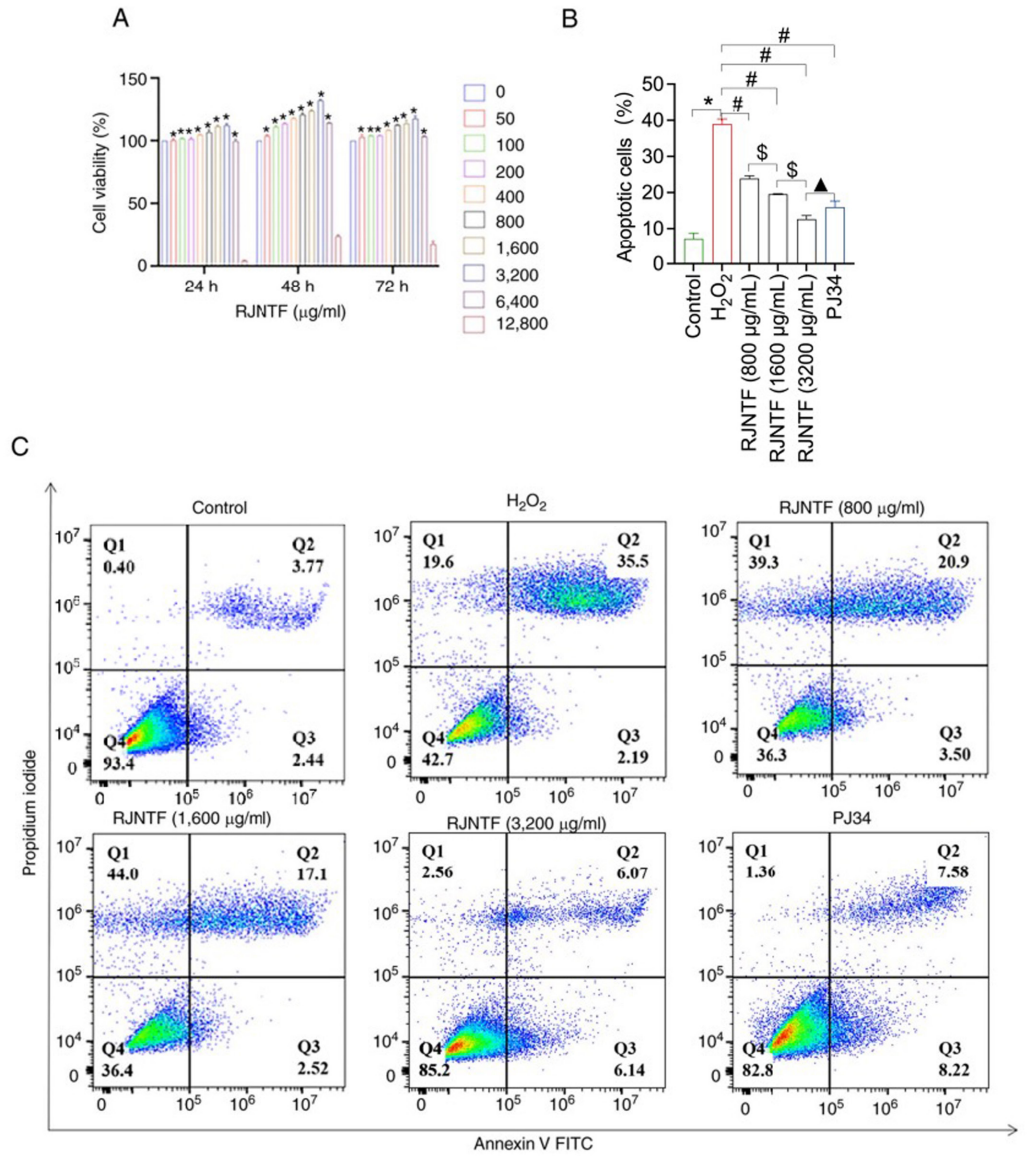
RJNTF inhibits chondrocyte apoptosis by regulating the PARP1/AIF pathway. (A) Chondrocytes were incubated for with different concentrations of RJNTF for 24, 48, or 72 h. Cell viability was assessed using a Cell Counting Kit-8 assay. (B) Analysis of the chondrocyte apoptosis rate in the different groups. (C) Apoptotic rate of chondrocytes in the different groups. *P<0.05 vs. control group; ^#^P<0.05 vs. H_2_O_2_ group, ^$^P<0.05 vs. RJNTF group, ^▲^P<0.05 vs. 3,200 µg/ml RJNTF group. PARP1, poly [ADP-ribose] polymerase-1; AIF, apoptosis inducing factor; RJNTF, Rongjin Niantong Fan; H_2_O_2_, hydrogen peroxide.

**Figure 10.**
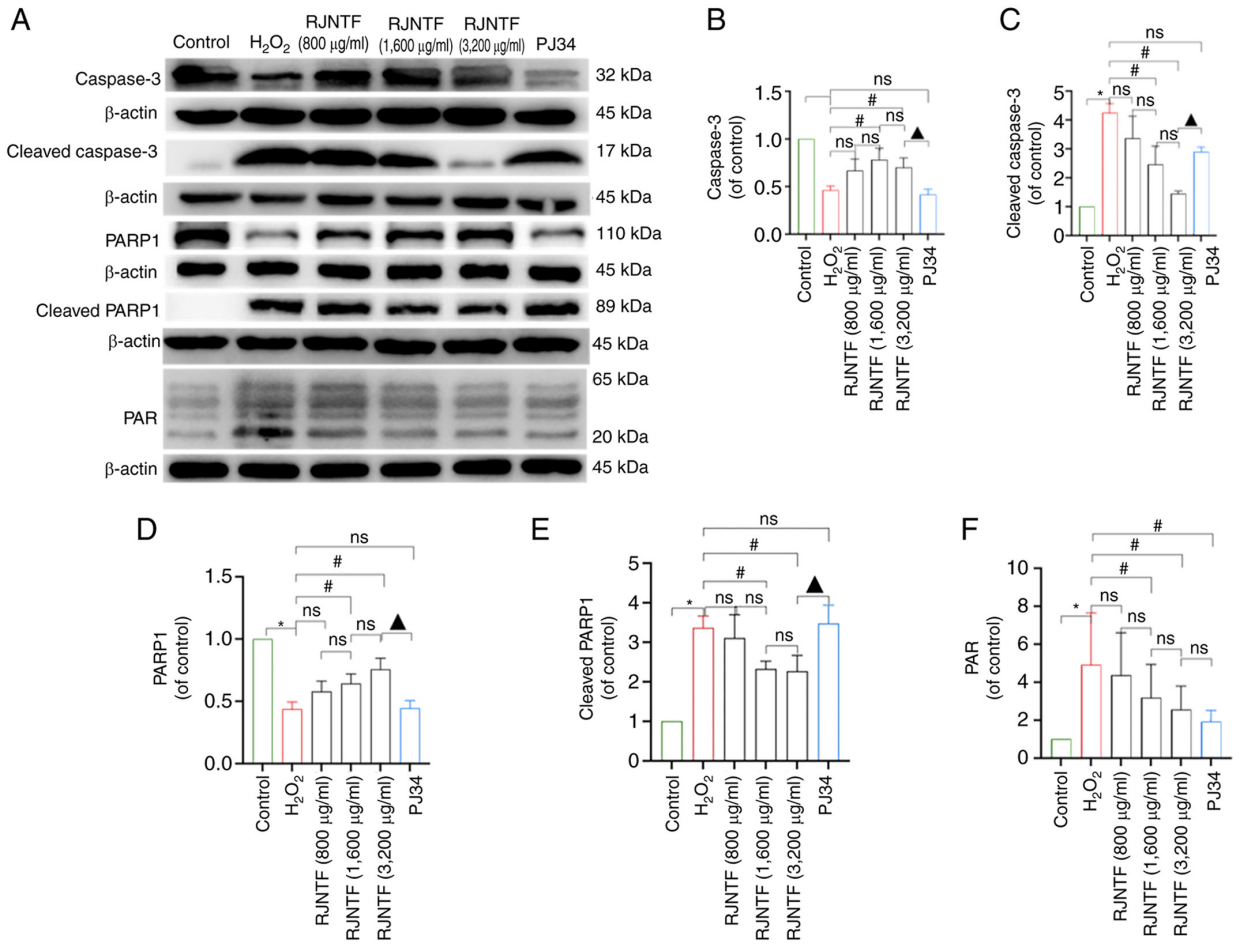
Effect of RJNTF on the expression of PARP1/AIF pathway related proteins. (A) The protein expression levels of caspase 3, cleaved caspase 3, PARP1, cleaved PARP1, and PAR in the chondrocytes in the different treatment groups were determined. Quantitative analysis of the relative protein expression levels of (B) caspase 3, (C) cleaved caspase 3 (D) PARP1, (E) cleaved PARP1 and (F) PAR. *P<0.05 vs. control group; ^#^P<0.05 vs. H_2_O_2_ group; ^ns^P<0.05 vs. RJNTF group;^▲^P<0.05 vs. 3,200 µg/ml RJNTF. PARP1, poly [ADP ribose] polymerase 1; PAR, poly ADP ribose; AIF, apoptosis inducing factor; H_2_O_2_, hydrogen peroxide.

